# Mortality in Hip Fracture Patients During the COVID-19 Pandemic: A Retrospective Analysis in a District General Hospital in the United Kingdom

**DOI:** 10.7759/cureus.27747

**Published:** 2022-08-07

**Authors:** Bakhat Yawar, Callum Auld, Jennifer Salmon, Ali Yawar, Mohammad Noah Khan, Hassan Abdulrahman, Adriana Sapumohotti, Eimhear Duffy, Seanna Meehan, Aoife McSorley, Curtis Neely, Ryan Flynn, Hushil Sandhu, Sami Mustafa, Ammal Imran Qureshi, Ayeisha Asim, Andrew McAdam, Brian Hanratty

**Affiliations:** 1 General Surgery, The Western Health and Social Care Trust (HSCNI), Derry/Londonderry, GBR; 2 Trauma and Orthopaedics, The Western Health and Social Care Trust (HSCNI), Derry/Londonderry, GBR; 3 Trauma and Orthopaedics, Royal Victoria Hospital, Belfast, GBR; 4 General Surgery, The Western Health and Social Care Trust (HSCNI) (Altnagelvin Area Hospital), Derry/Londonderry, GBR; 5 Urology, The Western Health and Social Care Trust (HSCNI), Derry/Londonderry, GBR; 6 Surgery, Worthing Hospital, Southampton, GBR; 7 Geriatrics, The Western Health and Social Care Trust (HSCNI), Derry/Londonderry, GBR

**Keywords:** covid 19 and hip fracture, fragility hip fracture, mortality in hip fracture, nottingham hip fracture score, geriatric hip fracture

## Abstract

Introduction

Hip fracture is commonly seen in elderly patients because of low-energy trauma. It carries significant morbidity and mortality. Scoring systems such as the Nottingham hip fracture score (NHFS) have shown a good correlation with increased mortality as the value of these scores increases. In our study, we aim to ascertain the hip fracture mortality in our population, compare the mortality in hip fractures compared to previously reported figures in literature and nationally reported figures during the first year of the COVID-19 pandemic, and also ascertain the usefulness of NHFS in predicting mortality in hip fractures.

Methods

We gathered mortality data on hip fracture patients admitted to our unit from January 1, 2020 to December 31, 2020. NHFS was calculated for all patients and the 30-day mortality rate was compared to previously reported hip fracture mortality rates using the standard mortality ratio (SMR). One-year mortality was stratified by placing patients in high and low NHFS groups. The log-rank test was used to compare hip fracture survival at one month and at one year in the high NHFS (NHFS >4) group and low NHFS group (NHFS value 4 or below). Additionally, a log-rank test was used to compare one-month and one-year survival in hip fractures managed with hemiarthroplasty, dynamic hip screw and intramedullary nail.

Results

In 2020, 388 patients were admitted with hip fractures to our unit. The crude mortality rate was 3.9% at 30 days and 20.88% at one year. Compared to the National Hip Fracture Database report for 2020, the incidence risk ratio for mortality was 0.46 (p-value<0.05). The SMR at 30 days was 0.34 (CI=0.17-0.51) and the SMR at one year was 0.63 (CI=0.49-0.77). The survival rate was higher at 30 days and one year in the low NHFS group compared to the high NHFS group (p-value<0.01). The survival rate at one month and one year were similar in groups managed with hemiarthroplasty, dynamic hip screws, and intramedullary nails (p-value>0.05).

Conclusions

Hip fracture mortality has been decreasing steadily and we noted a lower rate of hip fracture mortality compared to figures reported previously as per NHFS studies even though the study was conducted during the COVID-19 pandemic period. We also noted lower 30-day mortality in our hospital as compared to the national 30-day mortality rate for hip fracture patients in 2020.

## Introduction

Hip fracture is a serious injury seen more commonly in elderly patients. It has a significant financial burden on health services [1,2]. In the UK around 75,000 hip fractures are reported annually [1,2]. Most hip fractures are reported in patients over the age of 70 [2]. The total annual cost of managing incident hip fractures (i.e., during acute admission) in the UK has been reported to be £1.1 billion and the estimated overall annual UK expenditure on hip fractures is reported to be approximately £2 billion to £3 billion [3].

The National Hip Fracture Database (NHFD) is a national quality improvement project which aims to improve services provided to hip fracture patients. The NHFD reported a 30-day hip fracture mortality rate of 8.3% in 2020 compared to 6.5% in 2019 [4]. The rise was attributed to the impact of the COVID-19 pandemic. COVID-19 infection has been reported as an independent risk factor for increased mortality in hip fracture patients [5].

The primary objective of our study was to determine the 30-day and one-year mortality in our centre from January 1, 2020 to December 31, 2020 during the first year of the COVID-19 pandemic. We compared our 30-day mortality data with the figures reported by NHFD for the same year. Additionally, we compared our survival rate at 30 days and one year to previous data from the Nottingham studies which led to the development of Nottingham Hip Fracture Scores (NHFS) [6-8]. We calculated the 30-day and one-year survival rates in patients managed operatively with different surgical modalities. Furthermore, we calculated the survival rates in patients with extracapsular and intracapsular fractures.

## Materials and methods

Study design

This was a retrospective cohort study that included all hip fracture patients admitted to Altnagelvin Area Hospital from January 1, 2020 to December 31, 2020. Data were collected on a secure excel sheet on the hospital intranet. The study was registered with the local Audit and Quality Improvement Department. A formal ethics approval or Institutional Review Board approval was not required as per guidelines from National Health Service Health Research Authority (NHS HRA) regulations.

Inclusion criteria

All adult patients who sustained a hip fracture were admitted to our unit, and operated on in our hospital were included in the study.

Exclusion criteria

Patients who were included in our local NHFD file but were not classified as hip fractures were excluded from the study. These included periprosthetic fractures, femoral shaft fractures, distal femoral fractures, and isolated greater trochanter fractures. Paediatric patients were also excluded from the study.

Data collection

A database on all fractures reportable for NHFD is maintained by specialist nurses in our trust which was used to identify all patients presenting with hip fractures as per the inclusion criteria. Demographic data such as age, gender, usual place of residence (such as own home or institutionalised living, e.g., residential or nursing home), abbreviated mental test score (AMTS), and co-morbidities were available from this database. The Northern Ireland Electronic Care Record (NIECR) was used to check the haemoglobin level (Hb) at admission and the presence of active malignancy in the last 20 years before fracture. The above data were used to calculate the NHFS for all patients. NIECR was also used to identify patients who had died within 30 days and within one year after sustaining the fracture. NIECR also helped to identify the type of fracture sustained and the treatment modality, i.e., the operation performed for all patients.

Definitions and data variables collected

While collecting the demographic data, patients were stratified into age groups of <65, 65-85 and >85 years as per the NHFS to calculate the scores. AMTS ranged from 0 to 10 as per admission notes. Co-morbidities were classified as cardiovascular (including ischaemic heart disease, congestive heart failure, atrial fibrillation, previous myocardial infarction, hypertension, cardiomyopathy, bundle branch block, peripheral vascular disease), respiratory (including asthma, chronic obstructive pulmonary disease, bronchiectasis, lung fibrosis, etc), renal (including nephropathy, chronic kidney disease, etc.) and endocrine (including diabetes, Addison’s disease, Cushing’s disease, hypothyroidism, hyperthyroidism, etc.). Co-morbidities will not be described separately in the results, but these were used to formulate the final NHFS for the patients. After a review of NIECR, squamous cell carcinoma and basal cell carcinoma were not considered malignancy in the last 20 years for calculation of the NHFS score. NIECR was reviewed to ascertain 30-day and one-year mortality in all patients included in the study. The fractures were broadly classified as extracapsular (which includes intertrochanteric and subtrochanteric fractures) and intracapsular (which includes transcervical, basicervical and sub-capital fractures). Operative modalities considered for comparison of 30-day and one-year survival rates included hemiarthroplasty, intramedullary nail (IMN) and dynamic hip screw (DHS) whereas patients managed with cannulated hip screws (CHS), total hip replacement (THR) and non-operative management were excluded from this analysis. This is because patients managed with CHS and THR are usually younger and highly independent whereas the majority of patients managed with non-operative management are extremely frail and moribund [9-11]. In addition, for analysing one-year survival outcomes, patients were allocated to high and low NHFS groups (high NHFS=5 or above, low NHFS=4 or below).

Data analysis

Crude 30-day and one-year mortality rates were first calculated. The 30-day mortality rate was then compared to the mortality rate reported by NHFD for the same year using the incidence risk ratio. Thereafter, the 30-day and one-year mortality rates were compared to the figures reported by the Nottingham studies [6,7]. For the 30-day mortality rate, the standardised mortality ratio (SMR) was calculated based on mortality rates for the actual NHFS. The 30-day mortality rate was calculated for each NHFS score category and compared to 30-day mortality as reported by the Nottingham study i.e., new NHFS in 2012 [6]. For one-year mortality, SMR was calculated after dividing the patients into high and low NHFS groups and compared to one-year mortality reported by Wiles et al. [7]. Using a log-rank test, 30-day and one-year survival within our study population were compared based on high and low NHFS scores (to validate the usefulness of the NHFS), type of operation performed (hemiarthroplasty versus IMN versus DHS) and type of fracture (Extracapsular versus intracapsular).

## Results

Patient demographics

Around 388 patients were admitted with hip fractures over the period of our study. Most patients included in our study were elderly. Females were almost twice as likely to sustain a hip fracture compared to males. The majority of patients were admitted from their own homes. Basic demographic data based on age, gender, AMTS, residence, type of fracture sustained, and type of management offered to patients is detailed in Table 1.

**Table 1:** Baseline characteristics and demographics of patients admitted with hip fractures AMTS: abbreviated mental test score, DHS: dynamic hip screw, CNS: cannulated hip screws, IMN: intramedullary nail, THR: total hip replacement

Variable	Category	Value
Age (years)	Mean +/- standard deviation	78.19 +/- 11.45
	Median	81
	Range	30-97
Gender	Male	120 (30.9%)
	Female	268 (69.1%)
AMTS	Less than 7	98 (25.2%)
	7 or above	290 (74.8%)
Usual place of residence	Own home	334 (86.1%)
	Institutionalised	54 (13.9%)
Type of Fracture	Extracapsular	124 (31.9%)
	Intracapsular	264 (68.1%)
Type of management	Hemiarthroplasty	198 (51.0%)
	DHS	94 (24.2%)
	CNS	6 (1.5%)
	IMN	59 (15.3%)
	THR	23 (5.9%)
	Non-operative	8 (2.1%)

Crude 30-Day Mortality

15 patients died in the first 30 days after sustaining a hip fracture with a crude 30-day mortality rate of 3.9%. This was much lower compared to the 30-day mortality rate of 8.3% reported by NHFD for the year 2020 which recorded data from 173 UK hospitals and included 63284 patients [1]. The incident risk ratio was calculated to be IRR= 0.48 (p-value<0.01).

Crude One-Year Mortality

In our study, 81 patients died within one year of sustaining a hip fracture with a mortality rate of 20.88%.

SMR at 30 Days

Our mortality data was compared to the 30-day mortality data from the Nottingham study by Moppet et al. [6] and the calculated SMR was 0.34 (CI=0.17-0.51). The 30-day mortality rate in our study was much lower compared to the Nottingham study which was used to validate the NHFS. Table 2 sheds light on our 30-day mortality, the expected number of deaths as per NHFS data [6] and the mortality rate based on NHFS.

**Table 2:** NHFS predicted deaths and actual deaths in our study for 30-day mortality. NHFS: Nottingham Hip Fracture Score

NHFS	NHFS predicted 30-day mortality (%)	Study population size per NHFS (n)	Expected deaths	Observed deaths (o)	Percentage of deaths (o/n)
0	0.7	10	0.11	0	0%
1	1.1	23	0.391	1	4.30%
2	1.7	22	0.594	0	0%
3	2.7	21	0.924	0	0%
4	4.4	86	5.934	3	3.50%
5	6.9	95	10.45	1	1.05%
6	11	81	12.96	8	9.90%
7	16	42	10.08	1	2.40%
8	24	6	2.04	1	16.70%
9	34	2	0.9	0	0.00%
10	45	0	0	0	0.00%
			Total= 44.383	Total=15	

SMR at One Year

At one year the SMR was calculated to be 0.63 (CI=0.49 to 0.77). This was calculated after comparing our data to the one-year mortality data reported by the Nottingham study by Wiles et al. [7]. Table 3 explains the predicted one-year mortality based on the Nottingham study, the expected number of deaths, and observed a number of deaths and the observed number of deaths used for this analysis.

**Table 3:** NHFS predicted one-year mortality versus actual mortality in our study (patients have been categorised based on low and high NHFS). NHFS: Nottingham Hip Fracture Score

NHFS category	Predicted 1 year mortality (%)	Number of patients in our study	Expected number of deaths	Observed number of deaths
Low risk (=4)	15.9	162	25.758	14
High risk (>4)	45.5	226	102.83	67
			Total= 128.588	Total= 81

The 30-Day and One-Year Survival Rate Based on NHFS

A log-rank test was conducted to compare the survival in groups designated as high and low NHFS which showed a higher survival rate in the low NHFS group at one year but similar survival rates at one month (30 days). This result was statistically significant with a p-value<0.01. Further details regarding the analysis are given in Table 4.

**Table 4:** The 30-day and one-year survival data for hip fracture patients stratified as per NHFS NHFS: Nottingham Hip Fracture Score, Dt: number of events during the period, Ct: number of censored results during the period, Nt: number of surviving patients at the start of the period, St: the survival rate at the end of the period, S.Et: the standard error in measurement of the survival rate.

Time (months)	Events (Dt)	Censored (Ct)	Nt	Survival rate (St)	Standard error (S.Et)	Confidence interval		
						Lower	Upper	
High NHFS group (score 5 or above)								
0	0	0	226	1	0	1		1
1	11	0	226	0.95	0.01	0.91		0.97
12	56	159	215	0.70	0.03	0.64		0.76
Low NHFS group (score 4 or below)								
0	0	0	162	1	0	1		1
1	4	0	162	0.97	0.01	0.94		0.99
12	10	148	158	0.93	0.02	0.86		0.95

**Figure 1:**
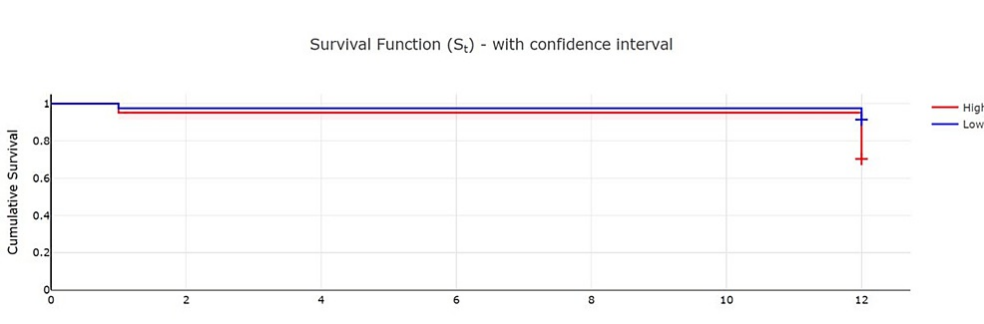
Kaplan Meier Curves demonstrating survival at one month and one year for high and low NHFS score patients NHFS: Nottingham Hip Fracture Score

In addition, Figure 1 shows the Kaplan Meier survival curves for both these groups.

The 30-Day and One-Year Survival Data Based on the Type of Operation

The 351 patients included in this analysis were managed with hemiarthroplasty, IMN and DHS. A log-rank test was used to compare survival in these patients after injury at 30 days (one month) and one year (12 months). The results showed no significant difference in survival rates at these timepoints in patients managed with these different operative modalities (p-value=0.82). Table 5 details these findings further.

**Table 5:** Survival data at one month and one year for patients undergoing different types of surgery Dt: number of events during the period, Ct: number of censored results during the period, Nt: number of surviving patients at the start of the period, St: the survival rate at the end of the period, S.Et: the standard error in measurement of the survival rate, DHS: dynamic hip screw, IMN: intramedullary nail

Time (months)	Events (Dt)	Censored Ct)	Nt	Survival rate (St)	Standard error (S.Et)	Confidence interval		
						Lower	Upper	
DHS								
0	0	0	94	1	0	1		1
1	5	0	94	0.95	0.02	0.88		0.98
12	17	72	89	0.77	0.04	0.67		0.84
Hemiarthroplasty								
0	0	0	198	1	0	1		1
1	2	0	198	0.99	0.01	0.96		0.99
12	39	157	196	0.79	0.03	0.73		0.84
IMN								
0	0	0	59	1	0	1		1
1	3	0	59	0.95	0.03	0.85		0.98
12	10	46	56	0.78	0.05	0.65		0.87

**Figure 2:**
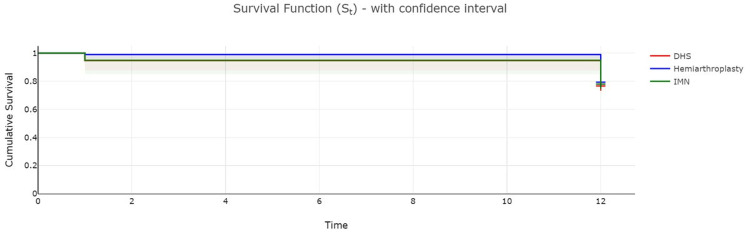
Kaplan Meier Curves demonstrating one-month and one-year survival after different operations for hip fracture DHS: dynamic hip screw, IMN: intramedullary nail

Figure 2 shows the Kaplan Meier curves for these three groups.

The 30-Day and One-Year Survival Data Based on the Type of Fracture

A log-rank test was performed to determine the difference in survival at one month and 12 months in patients who sustained extracapsular versus intracapsular fractures. We found no significant difference in survival at both the timepoints (p-value=0.75). Further details regarding the analysis are given in Table 6.

**Table 6:** Demonstration of one-month and one-year survival data after different types of fractures. Dt: number of events during the period, Ct: number of censored results during the period, Nt: number of surviving patients at the start of the period, St: the survival rate at the end of the period, S.Et: the standard error in measurement of the survival rate.

Time (months)	Events (Dt)	Censored Ct)	Nt	Survival rate (St)	Standard error (S.Et)	Confidence interval		
						Lower	Upper	
Extracapsular								
0	0	0	124	1	0	1		1
1	6	0	124	0.95	0.02	0.89		0.98
12	21	97	118	0.78	0.04	0.70		0.84
Intracapsular								
0	0	0	264	1	0	1		1
1	9	0	264	0.96	0.01	0.94		0.98
12	45	210	255	0.80	0.02	0.74		0.84

**Figure 3:**
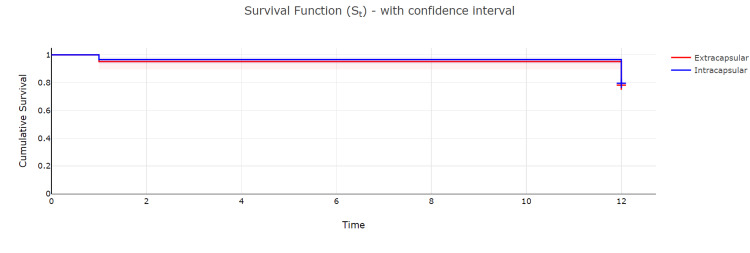
Kaplan Meier curves showing survival at one month and one year for different types of hip fractures.

Figure 3 shows the Kaplan Meier curves for these two groups.

## Discussion

Hip fractures are a common cause of admission to orthopaedics services and are defined as a fracture from the femoral head to 5 cm below the level of the lesser trochanter [12]. Hip fractures are associated with significant financial strain on health services due to the costs of hospitalisation, rehabilitation, medications, implants, and losses in productivity [13]. Our study aimed to look at the mortality associated with hip fractures as well as the scoring systems to predict mortality in hip fracture patients.

The 30-day and one-year mortality associated with hip fractures have been slowly decreasing over the last few decades. However, due to the COVID-19 pandemic in 2020, the 30-day mortality rate for hip fractures was reported to increase to 8.3% from a low of 6.5% in 2019 [1]. The trend of improvement in hip fracture mortality has been attributed to the introduction of guidelines by the National Institute of Clinical Excellence (NICE UK) [14] and the Best Practice Tariff for hip fracture management [15]. Despite this national trend of increase in hip fracture mortality in 2020, we noted that our 30-day hip fracture mortality rate remained low at 3.9%. One-year mortality from hip fractures has been reported between 15.1% and 29.3% in the literature [7,16,17]. In our study, we found that the one-year mortality was 20.88%, which was consistent with previously reported figures. It is unclear why the mortality rate (especially 30-day mortality) rate is lower in our trust compared to the national average. Our senior consultants report that the mortality rates in our hospital have historically been low although compared to figures reported nationally and regionally (i.e., from other hospitals in Northern Ireland). It is noteworthy that our Orthopaedics service follows the same guidelines followed by other hospitals in Northern Ireland for the management of hip fracture patients (namely NICE guidelines) [14]. One reason hypothesised by our consultants is that our hospital is geographically remote from other parts of the UK and Northern Ireland, has a high number of substantive and regular staff and has a high rate of staff retention. Due to this reason, there may be a better acquisition of skills to provide hip fracture care and more cohesiveness as a team. It would be interesting to perform further research into this phenomenon, and in the future, we aim to perform a regional survey to ascertain the knowledge of members of the multidisciplinary teams managing hip fracture patients in all hospitals in Northern Ireland to further investigate if our findings are supported by this hypothesis.

SMR has been described as a good indicator to evaluate a hospital’s mortality rate compared to other units providing the same service [18]. In our study, we noted that our SMR at 30 days and one year was much lower compared to the figures reported by previous studies [6,7] after accounting for the NHFS. The most plausible explanation for this is that these studies were performed approximately 10-12 years ago and since the introduction of NICE guidelines and Best Practice Tariff (BPT) [14,15], the management of hip fractures has improved leading to lower mortality which has been reported by NHFD as well [1]. Best Practice Tariff has introduced financial incentives for hospitals providing hip fracture care to ensure surgery within 36 hours of admission, orthogeriatrician review within 72 hours of admission, joint admission under orthopaedics and geriatric medicine and geriatrician-led rehabilitation [15]. Although the BPT is not currently implemented in Northern Ireland, all efforts are made in our hospital trust to adhere to these recommendations. The NICE guidelines also recommend surgery on the day of or the day after sustaining the hip fracture, sets criteria for types of implants used based on baseline mobility and fitness of patients, type of analgesia and anaesthesia, orthogeriatric assessment and early supported discharge from the hospital, etc. [14]. All efforts are made in our hospital trust to ensure the NICE guidelines are followed in the management of hip fracture patients. These guidelines have led to improved outcomes in hip fracture patients which has also been noted in our hospital.

We noted that the 30-day survival in low NHFS group patients was slightly lower than in the high NHFS group, but this did not reach statistical significance due to the overlap of confidence intervals. Previous studies performed with a much larger patient cohort have shown that the 30-day survival was indeed higher in patients with lower NHFS (with a score below 5) in patients who sustained a hip fracture or other femoral fractures [6,19,20]. For one-year mortality, our study did show a higher survival rate in patients with NHFS of less than 5, and these results were statistically significant, which was consistent with previously reported findings [7].

Our study has some limitations such as a small number of patients. Furthermore, these results are from a single centre which may not be consistent with other centres. In addition, although NICE guidelines are followed in Northern Ireland, the Best Practice Tariff is not legally applicable to hospitals in Northern Ireland when compared to the centres in England. However, our study does strengthen the previously noted findings that the NHFS remains a predictable tool to determine the mortality in hip fracture patients. It also sheds light on how the NHFS is overpredicting the actual mortality rates in hip fracture patients in the current era.

## Conclusions

Hip fractures continue to be a significant financial burden and the COVID-19 pandemic led to disruption in services leading to higher mortality in hip fracture patients nationally. However, we noted a low mortality rate in hip fracture patients during this period. Our study validates the usefulness of the NHFS in predicting the one-year mortality rates in hip fracture patients. However, we recommend that further large-scale national studies should be undertaken to validate and recalibrate the NHFS in predicting 30-day mortality in hip fracture patients.
